# Heritable Thrombophilia in Venous Thromboembolism in Northern Pakistan: A Cross-Sectional Study

**DOI:** 10.1155/2021/8317605

**Published:** 2021-10-25

**Authors:** Maria Khan, Chaudhry Altaf, Hamid Saeed Malik, Muhammad Abdul Naeem, Aamna Latif

**Affiliations:** ^1^Armed Forces Institute of Transfusion, Rawalpindi, Pakistan; ^2^CMH Medical College, Bahawalpur, Pakistan; ^3^Combined Military Hospital, Peshawar, Pakistan; ^4^Armed Forces Institute of Pathology, Rawalpindi, Pakistan

## Abstract

**Background:**

Venous thromboembolism (VTE) is referred to as formation of clots in a deep vein or lodging of thrombus towards the lungs which could be fatal yet preventable. The risk of developing VTE can be increased by various factors. Where there are innumerable acquired causes, the possibility of inherited thrombophilia cannot be ignored. In view of this, we have evaluated all patients with venous thromboembolism for inherited thrombophilia.

**Objective:**

To evaluate the frequencies of antithrombin (AT) deficiency, protein C and S deficiencies, Factor V Leiden, and prothrombin gene mutations in patients harboring venous thromboembolism.

**Materials and Methods:**

A study comprising of 880 patients who were presented with manifestations of venous thromboembolism was conducted from July 2016 to June 2017. A blood sample collected from patients was screened for thrombophilia defects encompassing AT, protein C and S deficiencies, Factor V Leiden, and prothrombin gene mutations. All acquired causes of thrombosis were excluded.

**Results:**

Of 880 patients who underwent screening for thrombophilia, 182 patients demonstrated VTE history. Their age ranged from 1 to 58 years. Males constituted a predominant group. About 45 (24.7%) patients had evidence of heritable thrombophilia. Of these, 20 (10.9%) had AT deficiency, 9 (4.9%) had Factor V Leiden mutation, 6 (3.2%) had protein C deficiency, whereas protein S deficiency and prothrombin gene mutation both were found in 5 (2.7%) patients.

**Conclusion:**

Our study illustrated the highest frequency of antithrombin deficiency among other investigated thrombophilia defects.

## 1. Introduction

Venous thromboembolism (VTE), a multifactorial condition which involves formation of blood clots either in a deep vein (deep vein thrombosis (DVT)) or thrombus fragments can dislodge into the lungs (pulmonary embolism (PE)), is associated with significant fatality rate [1]. An estimated 1-2 per 1000 individuals is affected annually with a reported number of deaths ranging from 60,000 to 100,000 [2, 3]. Although still unclear, remarkable progress has been made in recognizing and delineating the molecular and cellular processes which affect Virchow's triad in an interdependent manner. While venous stasis has been identified as the main consequential component among the three contributing factors for the occurrence of VTE, it alone cannot lead to thrombus formation and either of the two factors must be present for disease development. Circulatory stasis in combination with hypercoagulability has been proposed as being a more important and crucial determinant than endothelial injury for the occurrence of VTE [3, 4].

Where there are a number of known acquired risk factors, heritable thrombophilia represents a small but significant group which contributes to the pathogenesis of VTE by promoting hypercoagulability which is often presented as a moderate risk for VTE [4, 5]. The interaction of inherited and acquired risk factors is needed for the disease to manifest clinically and increased tendency towards thrombosis is seen in such patients [6, 7].

Heritable thrombophilia is characterized by the genetic predisposition to form clots. The inappropriate clotting results from mutations in the candidate genes of blood coagulation cascade such as protein C (PC) and protein S (PS), antithrombin (AT), prothrombin (PT), and Factor V Leiden (FVL). Another group encompassing rare genetic factors known to cause thrombosis include dysfibrinogenemia and hyperhomocysteinemia, while the third group includes a number of indeterminate factors such as elevated levels of factors VII, VIII, IX, and XI and plasminogen deficiency [7]. In the western population, the prevalence of heritable thrombophilia is 4% for prothrombin gene mutation and Factor V Leiden, followed by 0.3% for both protein C and protein S deficiencies and 0.03% for antithrombin deficiencies. Moreover, there is an inverse relation of the prevalence of these known causes with the relative risk of developing thrombosis [5]. However, conflicting results have been reported to establish whether heritable thrombophilia can predict disease recurrence [8–10].

Testing for heritable thrombophilia in patients with VTE has been a debatable issue and a number of guidelines have been developed in the setting of VTE for screening for heritable thrombophilia. These clinical situations include young patients with unprovoked VTE episodes, cases of recurrent VTE, patients with unprovoked VTE with either a strong family history of high risk thrombophilia or of VTE and thrombosis at unusual sites [11, 12].

Our study aimed at sharing our experience of determining the frequencies of protein C and S deficiencies, antithrombin deficiency, prothrombin gene mutation (F2G20210), and Factor V Leiden in patients with venous thromboembolism presenting at AFIP, a tertiary care setting.

## 2. Materials and Methods

A one year descriptive cross-sectional study was conducted at Armed Forces Institute of Pathology (AFIP) in the Department of Haematology from July 2016 to June 2017. Inclusion criteria consisted of all clinically and radiologically confirmed VTE cases of any age and gender and if they had first or recurrent VTE episode. All VTE episodes were considered unprovoked which can be defined as occurrence of VTE without exposure to any exogenous risk factor. Patients having comorbidities, VTE episode of <1 month duration, and arterial thrombosis and receiving anticoagulant treatment were excluded.

### 2.1. Procedure

The study was conducted after approval from the ethical committee. After informed consent, 3 ml of patients' blood samples was taken in tri-sodium citrate tubes for protein C and S, antithrombin, and FVL screening assays. For PCR of FVL and prothrombin gene mutation, a 3 ml sample was collected in ethylene diamine tetraacetate (EDTA) tubes. Platelet poor plasma was prepared after centrifugation at 4000 rpm for 15 minutes. Initial screening for deficiency of protein C and S was carried out using ProC Global reagent kit (Siemens Healthcare Diagnostics). It is based on the activation of endogenous protein C and intrinsic coagulation cascade by incubating test plasma with protein C activator and a contact phase. Activated protein C in conjunction with its cofactor protein S inhibits activated factors VIII and V and causes prolongation of clotting time called protein C activity-dependent clotting time (PCAT). PCAT is directly proportional to activity of protein C and protein S in test plasma. From this, normalized ratio was calculated and N.R of 0.69–>1.56 and PCAT 85–200 seconds were taken as normal. For those patients who had abnormal screening tests, protein C deficiency was detected by a functional chromogenic assay based on PROTAC activation method, taking 70–140% activity as normal, while protein S deficiency was detected by a coagulation-based assay taking 60–130% activity as normal. For Factor V Leiden screening test (activated protein C resistance), the patient's plasma was initially mixed with factor V deficient plasma in a ratio of 1 : 4 and the test was performed using ProC global kit. Normalized ratios of 0.86–1.10 and PCAT of 128–173 seconds were taken as normal. PCR for Factor V Leiden was performed on patients who had abnormal screening tests. Antithrombin deficiency was detected using a chromogenic functional assay performed on Sysmex CA-1500 automated coagulation analyzer with Siemens Healthcare Diagnostics kits, taking 75–125% activity as normal. The initial positive samples were retested by taking fresh blood sample and considered as positive if same results were found using the same assay. For PCR of both FVL and prothrombin gene mutation, DNA was extracted from peripheral blood samples by Chelex method. This was followed by DNA amplification using ABI 2700 thermal cycler (Applied Biosystems). The cycling conditions for FVL were denaturation at 95°C for 30 seconds, annealing at 60°C for 30 seconds followed by 30 cycles of extension at 72°C for 60 seconds, and final extension at 72°C for 3 minutes. Cycling conditions for prothrombin gene mutation were denaturation at 94°C for 60 seconds, annealing at 65°C for 60 seconds followed by 25 cycles of extension at 72°C for 90 seconds, and final extension at 72°C for 3 minutes. The amplified products along with heterozygous wild type and 100 bp ladder were subjected to 6% polyacrylamide gel electrophoresis at 200 volts for 25 minutes followed by staining with silver nitrate for adequate visualization.

### 2.2. Data Analysis

IBM SPSS version 20.0 was used to perform descriptive statistics and Pearson chi square test. Data have been stratified by age and gender to address effect modifiers. The association between variables was statistically significant at *p* value ≤0.05.

## 3. Results

A total of 882 patients were referred for thrombophilia screening during the study period. Out of these, 182 patients had clinical, radiological, and laboratory evidence of venous thromboembolism. The median age at diagnosis was 28 years. There were 125 (69%) males and 57 (31%) females with a male to female ratio of 2.2 : 1. Among these, 139 (76%) patients had a first episode of VTE, while 43 (24%) had a history of recurrent venous thrombosis. Presentations of patients presenting with VTE are given in Table 1.

**Figure 1:**
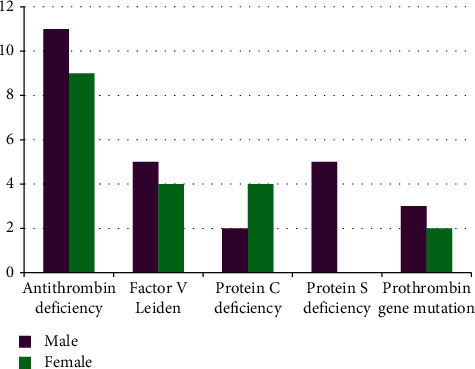
Gender distribution of heritable thrombophilia patients.

**Figure 2:**
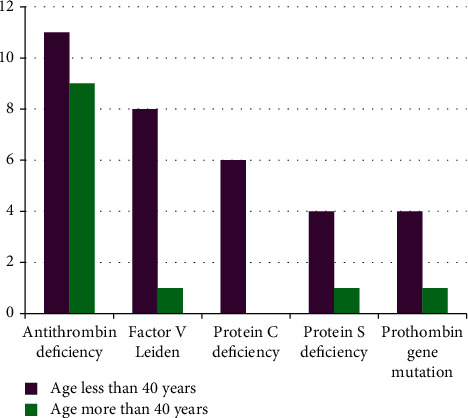
Age distribution pattern in heritable thrombophilia patients.

A total of 45 patients (24.7%) had evidence of heritable thrombophilia. The most common defect was antithrombin deficiency found in 20 (10.9%) patients followed by Factor V Leiden in 9 (5%) (1 had homozygous state and 8 had heterozygous state), protein C deficiency in 6 (3.2%), protein S deficiency in 5 (2.7%), and prothrombin gene mutation in 5 (2.7%) cases. The gender and age distribution patterns and clinical presentations of these patients are shown in Figures 1 and 2 and Table 2, respectively. It was found that the majority of the patients diagnosed to have a heritable cause of thrombosis had presented with a single episode of VTE, except for prothrombin gene mutation where there was a history of recurrent VTE in most of the cases. These data have been represented in Table 3.

## 4. Discussion

The interplay of genetic and various exogenous risk factors contributes to the etiology of VTE [10]. This interaction mostly leads to hypercoagulability severe enough to result in a disease phenotype [13]. Therefore, screening for heritable thrombophilia needs to be carried out in cases with a high index of clinical suspicion. Furthermore, the most important factors in thrombophilia testing are the timing of sample collection and interpretation of results. All relevant factors such as collection of sample during an episode of acute VTE or anticoagulant therapy were excluded for accurate results [14]. The current study illustrated the 24.7% frequency of heritable thrombophilia in VTE. Kim et al. reported a frequency of 18.3% in Korean population [15]. However, another Korean study by Kim et al. reported a frequency of 14.2% [16]. Interestingly, the most frequent thrombophilic defect in our patient was antithrombin deficiency, observed in 10.9% of cases. Lower frequencies have been reported in Korean studies of 4.2% and 3.9% [15, 16]. Mishra and Bedi reported a frequency of 6.4% for VTE in India [14]. Gu et al. observed a 2% prevalence of antithrombin deficiency in Chinese population [17]. The highest frequency of AT deficiency in our cohort of patients can be due to the regional variation in the prevalence of this inherited defect as well as testing methodology. However, genetic background of AT deficiency could not be determined due to unavailability of molecular assays in our settings and this is also limitation of our study. About 5% patients demonstrated mutation in FVL which is in sharp contrast to the findings of Taiwanese study which reported no case of FVL mutation [18]. In contrast, Kreidy reported a much higher frequency of 13% in a Lebanese study [19]. Earlier, Leroyer et al. from France also reported a high frequency of 14.5% [20]. In an Iranian study, 35.8% of the total patients presenting with VTE were positive for FVL mutation with 26.9% being heterozygous and 8.9% as homozygous [21]. This can be explained on the basis of the high prevalence of Factor V Leiden in these populations as well as the increased sensitivity of the testing techniques.

We reported a frequency of 3.2% for protein C deficiency, which is comparable to the results by Mishra and Bedi of 4.8% [14]. Gu et al. also observed a higher frequency of 8% in his study group [17]. Higher frequencies were observed in other studies of 7.7% and 7.2% [14, 16].

Protein S deficiency was observed in 2.7% patients in our study population. This is comparable with the frequency reported by Kim et al. of 2.8% [16]. Higher frequencies were reported in other studies, i.e., 16.6% and 13.7% [14, 15].

We reported a frequency of prothrombin gene mutation of 2.7%. Most of these patients were presented with a second episode of VTE. Yilmaza and Gunaydin reported a comparable frequency of 3.33% in Egyptian patients [22]. A much higher frequency was observed in Turkish population by Attia et al. [23]. All these differences in the results are attributable to the geographical diversity in the genetic distribution pattern of the disorder.

Another important observation in our data was that the patients with heritable thrombophilia presenting with VTE were found in younger age group of <40 years. This finding is in accordance with the international data [16].

Majority of the patients with heritable thrombophilia had presented after the first episode of VTE. Only in prothrombin gene mutation, the majority of cases had a history of recurrent episodes of venous thrombosis, particularly pulmonary embolism. This warrants investigation for prothrombin gene mutations in cases with more than one episode of VTE as well as pulmonary embolism.

## 5. Conclusion

Our study concludes that antithrombin deficiency followed by Factor V Leiden are the most common heritable causes of venous thromboembolism in our patients. Heritable thrombophilia is more frequently observed in the younger age groups and is very uncommon after 40 years of age. Hence, for all young patients presenting with unprovoked VTE, screening for heritable thrombophilia needs to be considered. This can aid the clinician in deciding the duration of anticoagulant therapy in different clinical settings.

## Figures and Tables

**Table 1:** Clinical presentations of VTE patients.

Presentation	Males	Females	Total
DVT lower limb	101	41	142
Pulmonary embolism	08	04	12
Venous sinus thrombosis	6	4	10
Portal vein thrombosis	5	5	10
Splenic vein thrombosis	—	2	2
Mesenteric vein thrombosis	2	—	2
Renal vein thrombosis	2	—	2
IVC thrombosis	2	—	2

**Table 2:** Clinical presentations of heritable thrombophilia patients.

	DVT	Pulmonary embolism	Venous sinus thrombosis	Portal vein thrombosis	Others
Antithrombin deficiency, *n* = 20	13	1	2	2	2
Factor V Leiden, *n* = 9	5	1	1	2	—
Protein C deficiency, *n* = 6	3	1	1	1	-
Protein S deficiency, *n* = 5	3	1	—	—	1
Prothrombin gene mutation, *n* = 5	2	3	—	—	—

**Table 3:** Frequency of heritable thrombophilia across the number of VTE episodes.

Defect	Single episode	Recurrent episodes
Antithrombin deficiency	14	6
Factor V Leiden	8	1
Protein C deficiency	5	1
Protein S deficiency	3	2
Prothrombin gene mutation	1	4

## Data Availability

All data supporting the findings of the current study are available within the submitted article.

## Abbreviations

VTE: Venous thromboembolism;
AT: Antithrombin;
FVL: Factor V Leiden.
